# Noninvasive models for the prediction of liver fibrosis in patients with chronic hepatitis B

**DOI:** 10.1186/s12876-024-03270-3

**Published:** 2024-05-24

**Authors:** Juanxia Wang, Xince Sun, Shibo Wei, Xinyue Chen, Haoyu Zhu, Youyou Liantang, Ruikun Bao, Yufeng Du

**Affiliations:** 1 Department of Infectious Diseases, Lanzhou University Second Hospital, No. 82 Cuiyingmen, Lanzhou, Gansu 730030 China; 2 The Second Clinical Medical College of Lanzhou University, Lanzhou, 730030 China; 3 Department of Epidemiology and Statistics, School of Public Health, Lanzhou University, No. 199 West Donggang R.D, Lanzhou, Gansu 730000 China

**Keywords:** Chronic hepatitis B, Liver fibrosis, Prediction, Noninvasive models.

## Abstract

**Objective:**

To evaluate the diagnostic accuracy of aspartate aminotransferase(AST)/ alanine transaminase (ALT), AST to platelet ratio index (APRI), fibrosis-4 score (FIB-4) and gamma-glutamyl transpeptidase to platelet count ratio (GPR) for hepatic fibrosis in patients with chronic hepatitis B (CHB).

**Methods:**

A total of 1210 CHB patients who underwent liver biopsy were divided into two groups: patients with no significant fibrosis (control group) and patients with significant fibrosis, and routine laboratory tests were retrospectively included. Logistic regression models were used for the prediction, and the area under the receiver operating characteristic (AUROC) was used to assess the diagnostic accuracy.

**Results:**

A total of 631 (52.1%) and 275 (22.7%) patients had significant fibrosis (≥ S2) and advanced fibrosis (≥ S3), respectively. The GPR showed significantly higher diagnostic accuracy than that of APRI, FiB-4, and AST/ALT to predict ≥ S2(significant fibrosis) and ≥ S3 fibrosis(advanced fibrosis), with an AUROC was 0.69 (95%CI: 0.66–0.71) and 0.72 (0.69–0.75), respectively. After stratified by the status of HBeAg ( positive or negative), GPR, APRI, and FiB-4 showed improved predicting performance for significant fibrosis and advanced fibrosis in HBeAg positive patients, with the most significant improvement was shown for GPR in predicting significant fibrosis (AUROC = 0.74, 95%CI: 0.70–0.78).

**Conclusions:**

Among the four noninvasive models, GPR has the best performance in the diagnosis of hepatic fibrosis in CHB patients and is more valuable in HBeAg-positive patients.

## Introduction

Hepatitis B virus (HBV) infection, a common chronic liver disease, has become a global health issue [1]. After viral infection, the host T cell immune response plays an important role in the clearance of HBV and is also an important mechanism for causing damage to hepatocytes [2, 3]. The repeated inflammatory damage and repair due to HBV infection can lead to liver fibrosis, cirrhosis and even hepatocellular liver cancer [4]. Hepatic fibrosis is the repair response of hepatocytes to chronic damage caused by viral infection, toxic damage, autoimmunity, and metabolic and genetic diseases, which leads to chronic deposition of extracellular matrix in the liver [5, 6]. The degree of liver fibrosis is one of the most important factors in determining whether patients with chronic hepatitis B (CHB) require active treatment and their prognosis. Therefore, the early diagnosis and intervention of CHB liver fibrosis are of great clinical significance. Currently, liver tissue biopsy is the ‘gold standard’ for the diagnosis and staging of liver fibrosis; however, the invasive nature and risk of bleeding limit its clinical application, and due to the requirements for the operator’s technical proficiency, it is difficult to widely promote its use in China, especially in resource-limited areas. Recently, a series of noninvasive liver fibrosis models have been developed to partially replace liver biopsy by combining several serological indicators; however, most are based on chronic viral hepatitis C cohorts and lack extensive clinical validation [7, 8]. These include the aspartate aminotransferase (AST)/platelet count (PLT) ratio index (APRI) and fibrosis score (FIB-4) [9, 10].

In this study, using liver histopathological findings and clinical data from 1210 HBV patients, four noninvasive liver fibrosis models with simple calculations and clinical operability, AST/ALT, APRI, FIB-4 and gamma-glutamyl transpeptidase (GGT) to PLT ratio (GPR), were selected and applied to the evaluation of liver fibrosis in patients with CHB to verify their diagnostic efficacy and provide a basis for the clinical selection of noninvasive liver fibrosis models.

## Materials and methods

### Study object

This study used retrospective analysis to include 1210 patients with CHB, aged 16–65 years, who were hospitalized at the Second Hospital of Lanzhou University for liver biopsy from October 2012 to December 2021. The diagnosis of CHB was in accordance with the Asia-Pacific Consensus on the Management of Chronic Viral Hepatitis B developed by the Asia-Pacific Society for the Study of the Liver [11], and the exclusion criteria for this study were: (1) co-infection with viruses other than HBV; (2) cirrhosis of the liver, drug-related liver injury, autoimmune hepatitis, alcoholic hepatitis, congenital hereditary metabolic liver disease, cholestasis, toxic liver disease, multi-organ failure, acute infection or trauma, and severe renal impairment; (3) liver malignancy or liver metastases from other systemic malignancies; (4) history of long-term smoking (> 10 year) and alcohol abuse (alcohol consumption > 140 g per week in female and > 210 g per week in male); (5) thyroid and other immune system diseases; (6) total parenteral nutrition; (7) history of chronic diseases such as hypertension, diabetes, heart valve disease, asthma, chronic obstructive pulmonary emphysema, and long-term medication (e.g., aspirin); (8) organ and bone marrow transplantation; (9) hepatoprotective therapy, immunomodulator therapy, and interferon or nucleoside (nucleotide) analogue antiviral therapy; (10) steatosis caused by alcohol, drug use, or cholestasis, among other causes.

### Laboratory testing

The height and weight of the study participants were measured using the SK-X80 electronic measuring instrument from Shuangjia Electronics. The patients were instructed to fast for 8 h. The following morning, 3 mL of fasting blood sample was collected to test routine blood indicators such as white blood cell count (WBC) and PLT. Non-invasive biomarkers were calculated [9, 10]: GPR, FIB-4 and APRI are calculated as follows: GPR = (GGT/upper limit of normal [ULN])/PLT (10^9^ /L) × 100, FIB-4 = [age (years) × AST (U/L)]/[PLT (10^9^/L) × ALT1/2 (U/L)], APRI = (AST/ULN)/PLT (10^9^/L) × 100. For routine blood tests, a fully automated hematology analyzer (Myriad 6800PLUS) was used. For liver function tests (liver enzymes, bilirubin levels, proteins levels) and for prothrombin time a fully automated biochemical analyzer (Beckman AU5800 and a fully automated coagulation analyzer (Wolfen Hemo CELL750) were utilized.

For HBeAg and HBsAg tests, fully automated chemiluminescence immunoassay analyzer ( Abbott Architect i) was employed. For HBV DNA tests, realtime fluorescence quantitative PCR (Roche Cobas CAP/CTM 96) was utilized, and the lower limit of detection is 20 IU/ml.The biopsy was completed within 24 h after blood testing.

### Histological pathological examination of the liver

All patients signed an informed consent form before liver biopsy. Liver tissue specimens were obtained using a 16G Bard biopsy needle to perform percutaneous liver biopsy after ultrasound localization. The specimen requirements were that the liver tissue was not less than 1.5 cm and contained more than six confluent areas. The specimens were fixed with 4% formaldehyde solution and routine paraffin sections were sent to the pathology department for the staging of liver fibrosis with reference to the Metavir scoring system [12] as follows: Stage S0: no fibrosis; Stage S1: increased fibrosis in the confluent area, but no fibrous septum formation; Stage S2: increased fibrosis in the confluent area, with little fibrous septum formation; Stage S3: numerous fibrous septa, but no sclerotic nodules; Stage S4: numerous fibrous septa, sclerotic nodules seen. Stages S0–S1 were defined as non-significant fibrosis and stages S2–S4 were defined as significant fibrosis. All specimens were reviewed sequentially by two experienced pathologists, and in case of disagreement, the review was repeated to reach a consensus.

### Statistical analysis

Normally distributed continuous variables were expressed using mean and standard deviation(SD), while those non-normally distributed were reported as median (25th–75th percentiles). Categorical variables were expressed as numbers and percentages. Differences in the characteristics of the non-significant fibrosis group and the significant fibrosis group were examined using the t-test or Mann-Whitney U test for continuous variables and χ^2^ test for categorical variables. Logistic regression models were used for the prediction, and the diagnostic accuracy of AST/ALT, APRI, FIB-4, and GPR for predicting fibrosis was assessed using the area under the ROC curves (AUROC), sensitivity, specificity, positive predictive values(PPV) and negative predictive values(NPV). Differences between the AUROCs were examined using the Z-test. Youden’s index was used to determine the optimal diagnostic cut-off value. In this study, further stratified analysis was performed according to whether HBeAg was positive or not, in which > 1 S/CO was used as the threshold value for determining HBeAg positivity. All tests were two-sided, and the difference was considered statistically significant at *P* < 0.05. SPSS (24.0) and R statistical software (3.4.0) were used for data analysis and ROC curve plotting.

## Results

### Comparison of general data

A total of 1210 cases enrolled, with 935(77.3%) cases in the non-significant hepatic fibrosis group (control group) and 275(22.7%) cases in the significant hepatic fibrosis group. As shown in Table 1, sex, BMI, drinking history, smoking history, the percentage of HBeAg-positive patients, and HBV DNA values were not statistically different between the two groups (*P* > 0.05), while age, prothrombin time (PT), ALT, AST, AST/ALT, GGT, APRI, FIB-4 and GPR were statistically different between two groups (*P* < 0.05).

### Predictive value of AST/ALT, APRI, FIB-4, and GPR in patients with different degrees of liver fibrosis

**Fig. 1:**
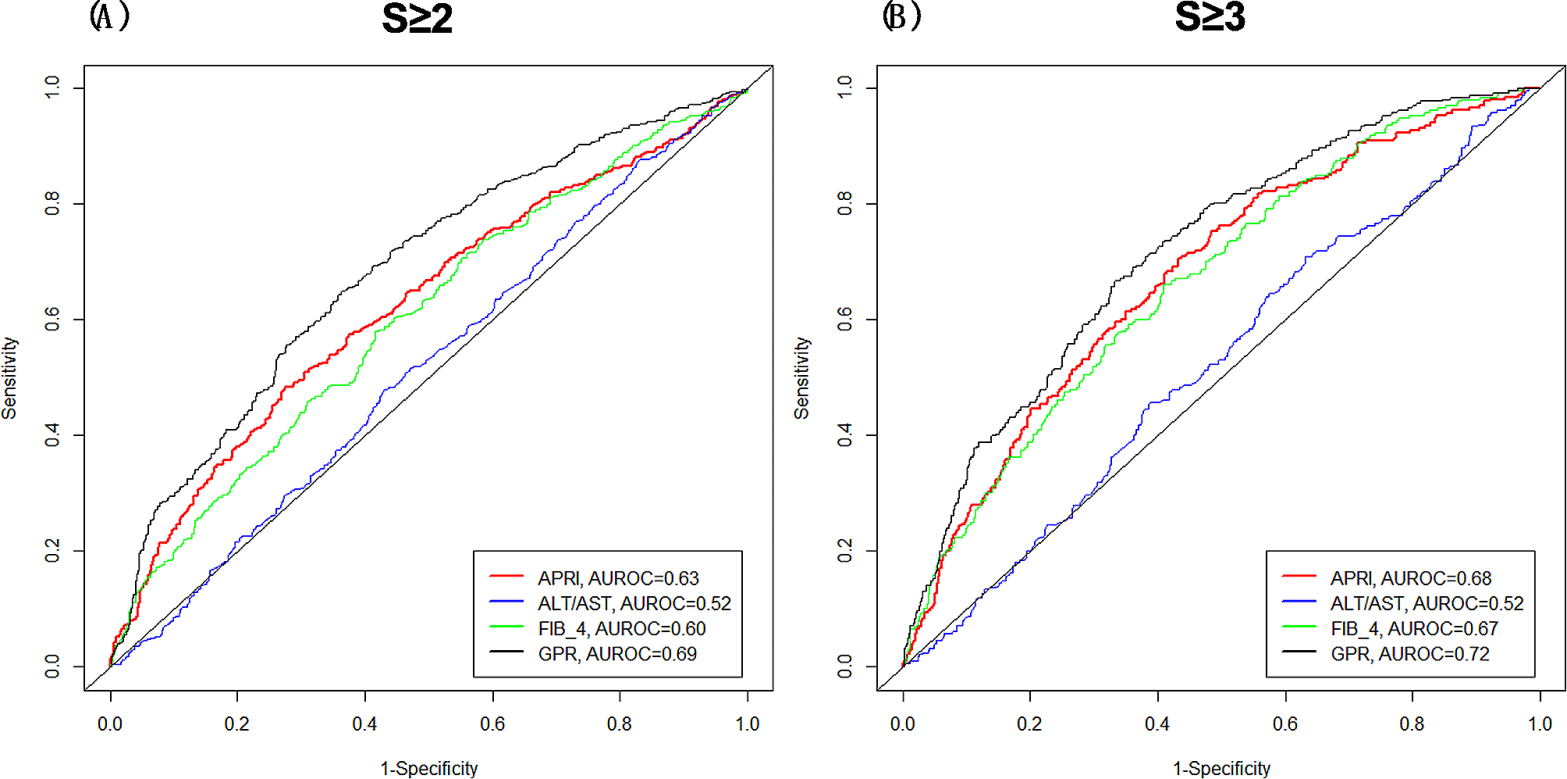
Predictive value of AST/ALT, APRI, FIB-4, and GPR for (**A**) significant fibrosis and (**B**) advanced fibrosis(the F3 and F4 were combined for statistical power consideration). *Abbreviations*: ALT, alanine transaminase; APRI, AST/PLT ratio index; AST, aspartate aminotransferase; AUROC, area under the receiver operating characteristic; FIB-4, fibrosis score; GPR, gamma-glutamyl transpeptidase to platelet count ratio

As presented in Table 2, the AUROCs of GPR, APRI, FIB-4 and AST/ALT for significant fibrosis (S2–S4) were 0.69 (95% confidence interval (CI): 0.66–0.71), 0.63 (95% CI: 0.60–0.65), 0.60 (95% CI: 0.58–0.63), and 0.52 (95% CI: 0.49–0.55), respectively. As for the advanced fibrosis (S3–S4), the AUROCs of GPR, APRI, FIB-4 and AST/ALT were 0.72 (95% CI: 0.69–0.75), 0.68 (95% CI: 0.65–0.70), 0.67 (95% CI: 0.64–0.70), and 0.52 (95% CI: 0.49–0.55), respectively. GPR, APRI and FIB-4 performed better for predicting advanced fibrosis than significant fibrosis. Compared with APRI, FIB-4 and AST/ALT, the GPR performed best in predicting advanced fibrosis and significant fibrosis (*P* < 0.05). The cut-off values, sensitivity, and specificity of AST/ALT, APRI, FIB-4, and GPR for predicting patients with stage S2–S4 and S3–S4 liver fibrosis are shown in Table 2. As the ROC curve shows, the AUROC of GPR in predicting advanced fibrosis and significant fibrosis is significantly larger than APRI, FIB-4 and AST/ALT (Fig. 1).

### Different predictive values of AST/ALT, APRI, FIB-4, GPR in HBeAg-positive and HBeAg-negative patients

Table 3 presents the results stratified by HBeAg status. Among HBeAg-positive patients, the AUROCs of GPR, APRI, FIB-4 and AST/ALT for significant fibrosis (S2–S4) were 0.74 (95% CI: 0.70–0.78), 0.66 (95% CI :0.62–0.70), 0.70 (95% CI: 0.65–0.74), and 0.53 (95% CI: 0.49–0.58), respectively. The AUROC of GPR and FIB-4 were not significantly different (*P* = 0.077). The GPR and FIB-4 performed better than APRI and AST/ALT in predicting advanced fibrosis, and the AUROCs were 0.75 (95% CI: 0.71–0.79) and 0.73 (95% CI: 0.69–0.77), respectively.

**Fig. 2:**
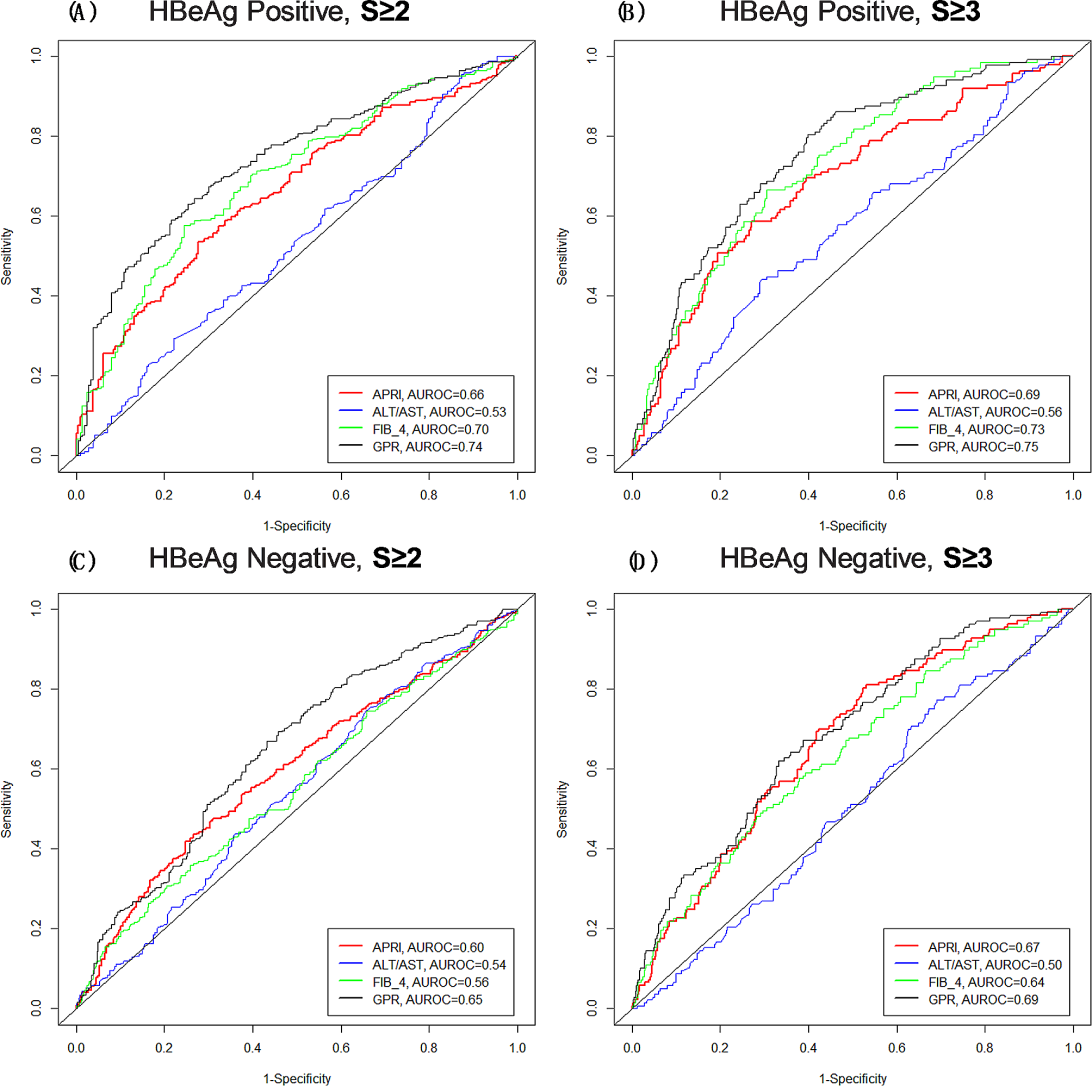
Predictive values of AST/ALT, APRI, FIB-4, and GPR for significant fibrosis and advanced fibrosis among (**A**, **B**) HBeAg-positive and (**C**, **D**) HBeAg-negative patients(the F3 and F4 were combined for statistical power consideration). *Abbreviations*: ALT, alanine transaminase; APRI, AST/PLT ratio index; AST, aspartate aminotransferase; AUROC, area under the receiver operating characteristic; FIB-4, fibrosis score; GPR, gamma-glutamyl transpeptidase to platelet count ratio

Of note, all four predictors exhibited lower diagnostic values for advanced fibrosis than significant fibrosis among HBeAg-negative patients. The GPR had a better fit than other three predictors in predicting significant fibrosis of HBeAg-negative patients, with an AUROC of 0.65 (95% CI: 0.61–0.68). As for the prediction of advanced fibrosis among HBeAg-negative patients, similar prediction accuracy was seen for GPR, APRI and FIB-4. Stratified by HBeAg status, the cut-off values, sensitivity, and specificity of four predictors in predicting stage S2–S4 and S3–S4 liver fibrosis are presented in Table 3. Figure 2 presents the ROC curves stratified by HBeAg status.

## Discussion

HBV infection has been the focus of clinical attention [13], and without timely and effective antiviral therapy patients with CHB can progress to cirrhosis or even hepatocellular carcinoma [14]. Monitoring the level of liver fibrosis in patients is essential for clinical management and prognosis judgment of patients with CHB. Currently, liver fibrosis is assessed primarily by pathological examination, imaging, serology and non-invasive model diagnosis. Liver biopsy is the gold standard for evaluating liver disease and assessing liver injury; however, it is an invasive operation with possible complications such as pain, bleeding, and infection, and the high cost of liver biopsy has prevented the full implementation of this technique in primary care hospitals; therefore, it has not been widely used in clinical practice. In order to overcome these limitations, much research has been carried out in the area of noninvasive fiber detection, including imaging evaluation such as transient elastography, shear wave elastography, and magnetic resonance elastography which are gradually being introduced into clinical practice [14–17]; however, due to the high cost of equipment, they are still not available in most primary hospitals in China. Therefore, compared to invasive and imaging-based tests, non-invasive fibrosis assessment based on serologic tests has been commonly used by Chinese scholars.

Fibroscan is a commonly used test for liver diseases in recent years, mainly evaluating the hardness and fat content of the liver. It has certain reference value for some chronic liver diseases, but it can not be widely carried out in primary hospital in China. Every patient can easily get a blood test, so the current common diagnostic models for the assessment of the degree of liver fibrosis in patients with CHB include APRI, FIB-4, and GPR are easier to be widely used [18]. Previous clinical reports have shown that these have good predictive diagnostic value for different stages of liver fibrosis, and APRI and FIB-4 are simple to calculate, and obtaining ALT, AST, and PLT is sufficient [18–21]. In this study, patients with CHB were divided into two groups based on the results of liver biopsy: patients with non-significant fibrosis (control group) and patients with advanced fibrosis. The results showed that there was a statistical difference between the two groups in age, PT, ALT, AST, AST/ALT, GGT, APRI, FIB-4 and GPR (*P* < 0.05). In the advanced fibrosis group, age, ALT, AST, AST/ALT, GGT, FIB-4 and GPR were significantly higher and PT was significantly lower than those in the control group, suggesting that the liver tissue in the significant fibrosis group was more severely damaged. We further analyzed the predictive diagnostic value of the different noninvasive indicators for different stages of liver fibrosis, and the AUROC for APRI was 0.63 for significant fibrosis and 0.68 for advanced fibrosis, which were higher than those for FIB-4 (0.60 for significant fibrosis / 0.67 for advanced fibrosis) and AST/ALT ratio (0.52 for both significant and advanced fibrosis). This is because APRI indicator is the ratios of AST and PLT, which can reduce the effect of abnormally elevated transaminases due to the active phase of hepatitis therefore APRI is of higher clinical value in evaluating the degree of liver fibrosis.

ALT and AST are among the most sensitive indicators of liver injury and are widely used in the evaluation of clinical liver function and the degree of injury. ALT and AST are mainly distributed in the hepatocyte cytoplasm and AST is significantly correlated with the necrosis of hepatocytes [22]. Xiyao et al. showed that in patients with primary hepatocellular carcinoma associated with hepatitis B with different liver disease bases, AST/ALT has a significant predictive value for disease changes [23].

The GPR model is a novel noninvasive assessment model for apparent cirrhosis in chronic HBV-infected patients proposed recently by Lemoine et al., and the degree of liver fibrosis was assessed using both GGT and PLT data.^24^ This study showed that the GPR had significant differences between advanced fibrosis group and control group, with significantly higher GPR was observed in advanced fibrosis group. This GPR demonstrates markedly superior predictive performance compared to other three noninvasive models in this study, with an AUC was 0.69 and 0.72 for significant fibrosis and advanced fibrosis, respectively. Our observed better performance of GPR was supported by three studies with smaller sample size than ours [23–26]. The performances of APRI and FIB-4 for the diagnosis of liver fibrosis is similar to our study [23–26]. However, other reports show that the performance of GPR is comparable to FIB-4 but a lower AUC for APRI [27], while another study [28] showed that the GPR did not show better diagnostic performance in predicting significant cirrhosis compared to APRI and FIB-4. The GGT is an important component of GPR and an important independent risk factor for liver fibrosis [29, 30]. The GGT levels are influenced by numerous factors such as the age and weight of the patient [31]. Difference in sample size, age and BMI of participants across studies may interpret this inconsistency. Our further analysis found that the AUC of the four noninvasive predictors, AST/ALT, APRI, FIB-4 and GPR, were significantly higher for HBeAg-positive than for HBeAg-negative AUCs, suggesting that the predictive efficacy of these predictors for HBeAg-positive patients was better than for HBeAg-negative patients and the predictive value of GPR was better than the other predictors. This result is also consistent with the natural course of patients with CHB, who have a short duration of immune tolerance, mild immune response, HBeAg positivity, and mild liver tissue inflammation and fibrosis [31–34]. In addition, statistical power may also interpret better prediction performance in HBeAg-positive patients, considering higher propotion of advanced fibrosis cases in HBeAg-positive patients (27.5%) than HBeAg-negative patients (19.4%) in our study.

A total of 1210 patients with CHB who underwent liver biopsy were included in this study, which has a large sample size, enabling a better assessment of the predictive efficacy of different noninvasive indicators. However, the limitation of this study includes the use of retrospective data, which may be subject to selection bias, and prospective studies are required to verify the reliability of the models constructed in this study. In addition, the cases in this study were from a single hospital, and the results need to be further validated in a multicenter study.

## Conclusions

In conclusion, GPR has better diagnostic efficacy than APRI, FIB-4, and AST/ALT for the staging of liver fibrosis in patients with CHB, the predictive value of GPR for HBeAg positivity is better than that for HBeAg negativity, and GPR is a better alternative to liver tissue biopsy. In clinical practice, dynamic assessment of GPR can help determine patient prognosis as well as guide clinical treatment options.

**Table 1:** Comparison of general information between the two groups of patients^a^

	Control group (*n* = 935)	Advanced fibrosis group (*n* = 275)	*P*
Age (year)	36.9±10.6	39.5±9.6	<0.001
Male	621 (66.4%)	181 (65.8%)	0.853
BMI (kg/m^2^)	22.5 (20.4, 25.1)	22.7 (20.5, 25.0)	0.699
Drinking	96 (10.4%)	33 (12.0%)	0.409
Smoking	242 (25.9%)	79 (28.7%)	0.340
Positive HBeAg	364 (38.9%)	138 (50.2%)	0.001
HBV DNA (log_10_IU/ml)	4.13 (2.91, 6.65)	5.04 (3.31, 6.36)	0.083
PT(10^9^/L)	157.0 (118.0, 193.0)	134.0 (98.0, 168.0)	<0.001
ALT (U/L)	30.0 (19.0, 49.0)	38.0 (26.0, 57.0)	<0.001
AST (U/L)	26.0 (20.0, 34.0)	33.0 (26.0, 45.0)	<0.001
AST/ALT	1.07 (0.92,1.36)	1.30 (1.08,1.67)	<0.001
GGT (U/L)	18.0 (13.0, 28.0)	28.0 (20.0, 50.0)	<0.001
APRI	0.43 (0.30, 0.67)	0.64 (0.44, 0.98)	<0.001
FIB-4	1.17 (0.76, 1.66)	1.58 (1.11, 2.39)	<0.001
GPR	0.25 (0.16, 0.41)	0.43 (0.28, 0.83)	<0.001

^a^ Continuous variables were expressed as mean ± SDs or median (inter-quartile range) and categorical variables as n (%). *Abbreviations*: ALT, alanine transaminase; APRI, AST/PLT ratio index; AST, glutamic oxaloacetic transaminase; BMI, body mass index; FIB-4, fibrosis score; GGT, gamma-glutamyl transpeptidase, GPR, GGT to PLT ratio; HBV, hepatitis B virus; PLT, platelet count; PT, prothrombin time.

**Table 2:** Predictive value of AST/ALT, APRI, FIB-4, and GPR for patients with different degrees of fibrosis

	AUROC (95% CI)	Cut-off	Sensitivity (%)	Specificity (%)	PPV	NPV	*P* compared to GPR
**Significant fibrosis(S2-S4)**							
GPR	0.69 (0.66–0.71)	0.28	0.65	0.64	0.66	0.63	1
APRI	0.63 (0.60–0.65)	0.56	0.48	0.73	0.66	0.56	<0.001
FIB4	0.60 (0.58–0.63)	1.23	0.58	0.58	0.6	0.56	<0.001
AST/ALT	0.52 (0.49–0.55)	0.83	0.48	0.57	0.55	0.5	<0.001
**Advanced fibrosis(S3-S4)**							
GPR	0.72 (0.69–0.75)	0.34	0.67	0.67	0.37	0.87	1
APRI	0.68 (0.65–0.70)	0.47	0.71	0.57	0.33	0.87	0.002
FIB4	0.67 (0.64–0.70)	1.29	0.66	0.59	0.32	0.86	0.007
AST/ALT	0.52 (0.49–0.55)	0.74	0.71	0.37	0.25	0.81	<0.001

*Abbreviations*: ALT, alanine transaminase; APRI, AST/PLT ratio index; AST, glutamic oxaloacetic transaminase; AUROC, area under the receiver operating characteristic; FIB-4, fibrosis score; GPR, gamma-glutamyl transpeptidase to platelet count ratio; NPV, negative predictive value; PPV, positive predictive value.

**Table 3:** Predictive values of AST/ALT, APRI, FIB-4, and GPR in HBeAg-positive and HBeAg-negative patients

	AUROC (95% CI)	Cut-off	Sensitivity (%)	Specificity (%)	PPV	NPV	*P* compared to GPR
**HBeAg-positive**							
Significant fibrosis (S2–S4)							
GPR	0.74 (0.70–0.78)	0.32	0.63	0.75	0.77	0.60	1
APRI	0.66 (0.62–0.70)	0.56	0.72	0.54	0.68	0.59	<0.001
FIB4	0.70 (0.65–0.74)	1.23	0.58	0.76	0.77	0.57	0.077
AST/ALT	0.53 (0.49–0.58)	0.48	0.96	0.13	0.60	0.71	<0.001
Advanced fibrosis (S3–S4)							
GPR	0.75 (0.71–0.79)	0.29	0.80	0.60	0.43	0.89	1
APRI	0.69 (0.64–0.73)	0.63	0.59	0.73	0.45	0.82	0.001
FIB4	0.73 (0.69–0.77)	1.28	0.67	0.70	0.46	0.85	0.279
AST/ALT	0.56 (0.52–0.61)	0.96	0.44	0.71	0.37	0.77	<0.001
**HBeAg-negative**							
Significant fibrosis (S2–S4)							
GPR	0.65 (0.61–0.68)	0.26	0.67	0.57	0.59	0.65	1
APRI	0.60 (0.56–0.63)	0.57	0.44	0.74	0.61	0.59	0.003
FIB4	0.56 (0.52–0.59)	1.90	0.31	0.80	0.59	0.55	<0.001
AST/ALT	0.54 (0.51–0.58)	1.11	0.34	0.75	0.56	0.55	<0.001
Advanced fibrosis (S3–S4)							
GPR	0.69 (0.65–0.72)	0.34	0.62	0.67	0.31	0.88	1
APRI	0.67 (0.63–0.70)	0.40	0.81	0.47	0.27	0.91	0.272
FIB4	0.64 (0.60–0.67)	1.67	0.50	0.70	0.29	0.85	0.076
AST/ALT	0.50 (0.47–0.54)	0.74	0.77	0.31	0.21	0.85	<0.001

*Abbreviations*: ALT, alanine transaminase; APRI, AST/PLT ratio index; AST, glutamic oxaloacetic transaminase; AUROC, area under the receiver operating characteristic; FIB-4, fibrosis score; GPR, gamma-glutamyl transpeptidase to platelet count ratio; NPV, negative predictive value; PPV, positive predictive value.

## Data Availability

The datasets used in the current study are available from the corresponding author on reasonable request.

## Abbreviations

AST: Aspartate aminotransferase
ALT: Alanine aminotransferase
PLT: Platelet
APRI: Aspartate aminotransferase to platelet ratio index
FIB-4: Fibrosis-4 score
GGT: Gamma-glutamyl transpeptidase
GPR: Gamma-glutamyl transpeptidase to platelet count ratio
CHB: Chronic hepatitis B
AUROC: Area under the receiver operating characteristic curve
HBV: Hepatitis B virus
WBC: White blood cell count
ULN: Upper limit of normal
FBG: Fasting blood glucose
UA: Uric acid
ROC: Receiver operating characteristic curves
BMI: Body mass index
PT: Prothrombin time
NPV: Negative predictive value
PPV: Positive predictive value

## Declarations

### Author details

^1^ Department of Infectious Diseases, Lanzhou University Second Hospital, Lanzhou 730030, China. ^2^ The Second Clinical Medical College of Lanzhou University, Lanzhou 730030, China. ^3^ Department of Epidemiology and Statistics, School of Public Health, Lanzhou University, Lanzhou 730000, China.
